# Improved ganoderic acids production in *Ganoderma lucidum* by wood decaying components

**DOI:** 10.1038/srep46623

**Published:** 2017-04-19

**Authors:** Yanru Hu, Shakeel Ahmed, Jiawei Li, Biaobiao Luo, Zengyan Gao, Qiyun Zhang, Xiaohua Li, Xuebo Hu

**Affiliations:** 1 Laboratory of Drug Discovery and Molecular Engineering, Department of Medicinal Plants, College of Plant Science and Technology, Huazhong Agricultural University, Wuhan, 430070 China; 2 National-Regional Joint Engineering Research Center in Hubei for Medicinal Plant Breeding and Cultivation, Huazhong Agricultural University, Wuhan, 430070 China; 3 Medicinal Plant Engineering Research Center of Hubei Province, Huazhong Agricultural University, Wuhan, 430070 China; 4 Biomedical Center, Huazhong Agricultural University, Wuhan, 430070 China

## Abstract

*Ganoderma lucidum* is a legendary Traditional Chinese Medicine (TCM) over a few thousands of years and one kind of its major active components are Ganoderic acids (GAs). GAs are largely produced in the mushroom primordium and fruiting body but much less in mycelium stage. However, little is known on the underlying regulatory mechanism. As a saprophytic fungus, *G. lucidum* solely obtains nutrients by wood decaying. Wood in general contains sophisticated chemical components with diverse structural units. To explore a strategy that extensively leads to GAs induction in the submerged liquid fermentation, all chemical components that might be possibly from the wood decaying were tested individually as GAs inducers. It was found that GAs production increased 85.96% by 1.5% microcrystalline cellulose (MCC) and 63.90% by 0.5% D-galactose. The transcription level of a few rate-limiting or chemically diverting enzymes responsible for GAs biosynthesis was greatly induced by MCC and D-galactose. The concentration and time-course titration study indicated that these two chemicals might not be utilized as carbon sources but they played a comprehensive role in the secondary metabolites synthesis. Our data indicated that MCC and D-galactose might be further industrialized for higher GAs production in *G. lucidum* in submerged fermentation.

*Ganoderma lucidum* is a medicinal fungus which is widely known as “Lingzhi” or “Reishi” in East Asia. Lingzhi has been used as a medical material for a few thousand years in China^1^. Recent study indicated that *G. lucidum* has many pharmacological activities such as antimicrobial, antiviral, antitumor, antiallergic, immunomodulating, anti-inflammatory, antiatherogenic and against obesity^2^,^3^,^4^,^5^,^6^,^7^,^8^. The most important bioactive products of *G. lucidum*, as claimed by numerous studies^9^,^10^,^11^,^12^,^13^,^14^,^15^,^16^, are Ganoderic acids (GAs), which belong to a class of oxygenated C30 lanostane-type triterpenoids (Fig. 1).

Production of GAs without tedious *G. lucidum* culture has been a target for decades because of huge market demand. GAs production from mycelium is much easier to control with fermentation technology, compared to the classical extraction from fruiting bodies. Medicinal metabolites may be extracted from mycelium without a long mushroom stage^17^. However, the mycelium of *G. lucidum* has a low content of GAs^18^. In order to increase the yield of GAs during fermentation, various strategies have been tried. In general, factors like culture medium, temperature, pH, oxygen and light who inevitably affect the growth of the fungi, may affect the GAs content^17^. A swapping flask shaking/static fermentation greatly improved the GAs yield^19^. Shifting temperature during submerged liquid fermentation also significantly improved the GAs production up to 37.11%^20^. Other studies focus on additives for the GAs induction, such as total GAs increased 30% by fungal elicitors treatment^21^, 45% by methyl jasmonate^22^, 105% by acetic acid^23^, 80% by aspirin^24^ and 90% by ethylene^25^. The static fermentation seemed to promote GAs yield higher, such as the GAs level increased 270% by Ca^2+^ activation^26^, while Mn^2+^ increased the GAs content by 220%^27^. In order to enhance the oxygen utilization of *G.lucidum*, mushroom transformed with *Vitreoscilla hemoglobin* gene showed up to two times higher GAs production over the non-transgenic control^28^. Nevertheless, the diverse treatments that brought to higher GAs production help to shape a better route towards the understanding of GAs synthesis and regulation. On the other hand, how these chemicals profoundly affecting the overall biological logic of the fungi is largely unknown. It also raises a safety issue with some of the treatments such as aspirin or heavy metals.

In most fungi and higher plants, GAs are synthesized via the mevalonate (MVA) pathway^29^,^30^. Most of the enzymes responsible for GAs have been elucidated (Fig. 1). Among these enzymes, three are important for the GAs biosynthesis, which are: 3-Hydroxy-3-methyglutaryl coenzyme A reductase (HMGR), a rate-limiting enzyme of the mevalonate/isoprenoid pathway; squalene synthase (SQS), an enzyme catalyzing the first step specific to the triterpenoids and lanosterol synthase (LAS), the enzyme responsible for the formation of lanostane-type triterpene skeleton^26^,^31^,^32^,^33^. By a series of further oxidation and reduction reactions, various GA derivatives are formed in current study as shown in Fig. 1.

*G. lucidum* and its close relatives obtain nutrients through wood decomposition, which results in extensive delignification of wood around the world^34^. Wood chemical composition mainly includes cellulose, hemicellulose and lignin. Cellulose is composed of glucose units through β-1, 4 glycosidic bond while hemi-cellulose is formed by a variety of simple sugars, such as D-galactose, L-arabinose, D-xylose, D-rhamnoseand D-mannose^35^,^36^,^37^. Lignin is another important structural material in the plant cell wall^38^. Lignin digestion by fungi is conducted by peroxidase or laccase, producing mainly three kinds of phenol elements: coniferyl alcohol, sinapyl alcohol and paracoumaryl alcohol^39^. The nutrients from the wood decay solely support *G. lucidum* grows from a spore to mycelium, then primordia and last into fruiting body.

In this study, inspired by a natural phenomenon that various tissues and organs of the fungus could grow out of simple wood, we tried to find out possible degradative components from wood that could be a significant spur for GAs biosynthesis that might be lent by us for liquid fermentation. In this regard, various components of cellulose, hemicellulose and lignin were tested for the total GAs production in the liquid fermentation. Our findings will benefit the future research in this economically as well as medicinally important mushroom.

## Results

### The effect of possible wood chemical degradation components on GAs yield in liquid fermentation of *G. lucidum*

In order to find out whether any possible chemical component from wood decomposition would significantly improve the GAs production of *G. lucidum* in liquid fermentation, we tested all possible chemical components of wood decomposition as a preliminary screening of the potential inducers. Chemicals tested for GAs induction were MCC and its degradative product D-cellobiose, lignin and its degradation product coniferyl alcohol and various hemicellulose degradation products, including D-galactose, L-arabinose, D-xylose and D-rhamnose, D-mannose, glucan hyaluronic acid and algin sodium mannuronic acid. The dry weight and total GAs for each treatment were measured, using potato dextrose agar (PDA) media culture as a control. The effect of different concentrations of the above chemicals was also tested (Fig. 2). In our hands, MCC could not be dissolved in any solvent, but it could be evenly dispersed in the culture media. As it is difficult to separate the MCC from mycelium through filtering, so we compare the GAs production in 100 ml medium. The result showed that the GAs production increased along with MCC dosage, while D-cellobiose had no effect on GAs biosynthesis and *G. lucidum* growth at a concentration of 0.1%, 0.5% and 0.25% (Fig. 2a–c). Though adding of L-arabinose and D-xylose increased the biomass of *G. lucidum* significantly, they had no contribution to GAs biosynthesis (Fig. 2d–f). On the other hand, D-galactose significantly induced GAs biosynthesis and it promoted *G. lucidum* growth with an increase of 58.9% at the dosage of 0.5% (Fig. 2d–f). It showed that 0.5% D-rhamnose also enhanced GAs accumulation by 22.4% (Fig. 2d–f). Lignin, another important compound of wood degradation, could increase GAs content significantly (Fig. 2g–i). Another wood degradation product, the coniferyl alcohol, produced no effect on GAs content. Additionally, we found comparably high triterpenoid background absorbance in lignin and coniferyl alcohol, which depreciated our interest on further optimization, especially with a low improvement by these two chemicals. The preliminary screening prompted us to further concentrate on MCC and D-galactose as the GAs inducers.

### Optimization of inducer dosage and timing in fermentation

In order to find out a suitable dosage for MCC and D-galactose in our study, different concentrations of these two chemicals were added into the fermentation medium on the third day of culture. As shown in Fig. 3, GAs production increased along with the dosage of MCC. The content increased significantly with 1.5% to 3.5% MCC (Fig. 3a). D-galactose also significantly induced GAs biosynthesis and promoted the growth of G. *lucidum* on the concentration of 0.25% and 0.5%, but lower or higher concentration of D-galactose resulted insignificance (Fig. 3b). Since MCC is insoluble in the culture media, a common dispersant, Tween-20 was added to increase the permeability. The data showed that tween-20 significantly increased the GAs yield (Fig. 3c). To check the possible synergic effect of MCC and D-galactose on the GA induction, different proportions of these two chemicals were added and tested on GAs production. Results proved that MCC and D-galactose at a ratio of 1:1, 1:2 or 1:3 all significantly increased GAs production, compared to the untreated control (Fig. 3d). However, GAs from the combination was not greatly higher than either galactose or MCC alone (Fig. 3b–d). We also tested adding of the inducer at an interval of every 2 days during the fermentation. The results indicated that addition of both MCC and D-galactose at the day 3 greatly stimulated the GAs yield (Fig. 4a,b). On the third day, the MCC leaded to GAs increase by 85.96% (from 11.4 mg/100 ml to 21.2 mg/100 ml) as shown in Fig. 4a and D-galactose resulted a GAs increase of 63.9% (from 12.42 mg/100 ml to 20.36 mg/100 ml) (Fig. 4b). It means the third day was the best time for adding of the inducer. In order to verify the fermentation time of MCC and D-Galactose on GAs induction, we recorded the dynamic change of GAs biosynthesis every 24 hrs for 4 days (Fig. 4c,d). The results demonstrated that GAs yield was significantly increased by MCC from day 1 to day 4, while the yield from D-galactose induction was significant higher than that of control from day 3 to day 4 (Fig. 4c,d).

### Transcript alterations responding to inducers

To further understand the impact of MCC and D-galactose addition on the metabolism, the expression of genes in GAs biosynthesis was investigated by qRT-PCR. Therefore, the transcriptional levels of GA biosynthetic genes HMGR, SQS and LAS in the fungi treated with 1.5% MCC or 0.5% D-galactose were detected. As shown in Fig. 5, as the upstream rate-limiting gene, HMGR expression level was high from the beginning 2 hrs to 24 hrs of the fermentation, and then declined from 48 hrs to 72 hrs. But the HMGR expression level recovered at 96 hrs. MCC shared the similar trend with the control but the expression level was significantly higher from 48 hrs to 96 hrs, with the highest expression of 3.5 fold than that of the beginning (Fig. 5a). On the other hand, D-galactose also induced HMGR’s expression but the peak was at the 24 hrs with a 4.3 fold increase (Fig. 5d).

The SQS expression reached the highest point at 6 hrs and then decreased to a much lower level in the rest 4 days. However, MCC promoted SQS expression at 6 hrs to an even higher level and it enhanced the expression at the day 3 and day 4 (Fig. 5b). D-galactose significantly enhanced SQS expression at day 1 and day 4 after the expression reached to the peak at 6 hrs (Fig. 5e).

The LAS gene expression gradually increased along the time while the gene’s expression was further increased by either the MCC or D-galactose. The difference between MCC and D-galactose is that MCC greatly induces the gene expression from day 2 to day 4 but D-galactose increases the expression from day 1 to day 2. This might be a valuable clue for further fermentation optimization.

## Discussion

### Wood chemical degradation components affect GAs biosynthesis in liquid fermentation of *G. lucidum*

*G. lucidum* has been used as a tonic TCM for a long history in Southeast Asia countries. The scarcity of the wild-grown mushroom made it extremely precious in old days. However, the recent in-depth biological study of *G. lucidum* has made the cultivation of the mushroom fairly straightforward^40^. However, cultivation of the mushroom need a large quantity of fine wood and the fruiting body is hard to be consumed because it is highly-lignified and it has a strong bitter taste with normal boiling. Consumers turn to take the spores of *G. lucidum* for an alternative. However, compared to mycelia and fruiting bodies, the biomass of the spores is very limited. Therefore, the medicinal applications of Lingzhi have to be more efficient, unless there are scientific proofs to support the advantage of spores over mycelia and fruiting bodies.

*G. lucidum* essentially belonging to a member of Basidiomycota (white-rot fungi), which have the ability to grow on wood for their growth^41^. *G. lucidum* is highly adoptable to grow on a dozen of lignocellulosic biomass materials due to its rich enzymes which can degrade different components of wood including cellulose, hemicellulose as well as lignin^42^. Recent genome sequencing revealed that *G. lucidum* has one of the largest sets of enzymes for wood decomposition in the Basidiomycota^18^. *G. lucidum* is fully dependent on wood decomposition for its growth nutrients but the mushroom undergoes a few developmental stages, namely, the mycelium, primordia and fruiting body and sporing stage during its life cycle. The mycelium is easy to culture in bulk yield with modern fermentation technology. However, it is reported that the GAs content was much less compared to that of primordia and fruiting body^18^. We hypothesized that there is a trigger of the morphologic change for *G. lucidum* and it could be a chemical from wood decomposition.

In this study, we tested all possible chemical components or units from wood decomposition as a preliminary screening of the potential inducers. MCC, a natural occurring polymer of glucose linked with 1–4 β glycosidic bonds, is from wood partial decomposition. MCC serves as an uncomplete state of the wood during its composition. The results indicated that GAs yield increased alone the culture time (Figs 2 and 3). However, the cellobiose, a disaccharide formed with 1-4 β glycosidic bonds, did not lead to GAs over production (Fig. 2). The difference might be because there are some chemical residues other than glucose in the MCC made a contribution to the GAs over production. The assumption is based on the fact that the glycosidic bonds in MCC and cellobiose are the same and so their final products as well.

Further tests were conducted with lignin and its degradation product coniferyl alcohol and various hemicellulose degradation products, including D-galactose, L-arabinose, D-xylose and D-rhamnose, D-mannose, glucan hyaluronic acid and algin sodium mannuronic acid (Fig. 2). Of all the chemicals tested above, only D-galactose significantly increased both the biomass and GAs yield. Other sugars had little improvement in the GAs production or even adversely affected its yield. Galactose is only 6% of the glucose and 3% of all monosaccharide in the pine tree^43^, indicating a very low ratio in the wood composition. Yeast evolved to metabolize galactose as a carbon source in case glucose is absent^44^. In our experiments, however, the glucose was sufficient for the whole stage of fermentation, and so it is less likely to have a similar genetic circuit in the *G. lucidum.* On the other hand, the biomass did not increase with the concentration of galactose (Figs 2 and 3), indicated that it was not a main carbon source to support the mushroom’s growth. Further study is needed to look into the mechanism underlying the improvement of the biomass and GAs content of *G. lucidum* by galactose.

### Optimization of GAs production

In a specific culture system, cell growth and metabolite yield are greatly affected by the optimization of fermentation time and the dosage of an elicitor^45^. Previous studies also proved that GAs yield was greatly influenced by a number of environmental factors including the elicitor, parameters of fermentation process and culture medium^21^,^46^,^47^,^48^.

In order to find the suitable addition dosage in our study, different concentrations of MCC and D-galactose were added into the fermentation medium on the third day of culture. The galactose with a certain concentration stimulated the GAs production (Fig. 3b), indicating it was not a carbon source, as discussed above. Dispersant Tween-20 was added to increase the permeability of MCC since it is not soluble in the media (Fig. 3c,d). It is worth noting that Tween-20 has inhibitive effect on GAs production without MCC addition (Fig. 3d), indicating that Tween-20 did assist MCC permeability.

As the secondary metabolites, triterpenoids essentially are a group of chemicals that are not necessary for basic growth but indispensable for the survival and competition in the environment^49^,^50^. So it may have a balance between optimal growth with rich nutrients and stressed growth with limited nutrient environment. In this regard, we sought to find a suitable addition time for the MCC and D-galactose. Our results proved that adding of both MCC and galactose at the third day of fermentation gained the highest GAs yield (Fig. 4). However, it is still unknown the particular role of MCC and galactose in GAs induction in the course of fermentation.

### Understanding of the GAs biosynthesis at molecular level

**Figure 1:**
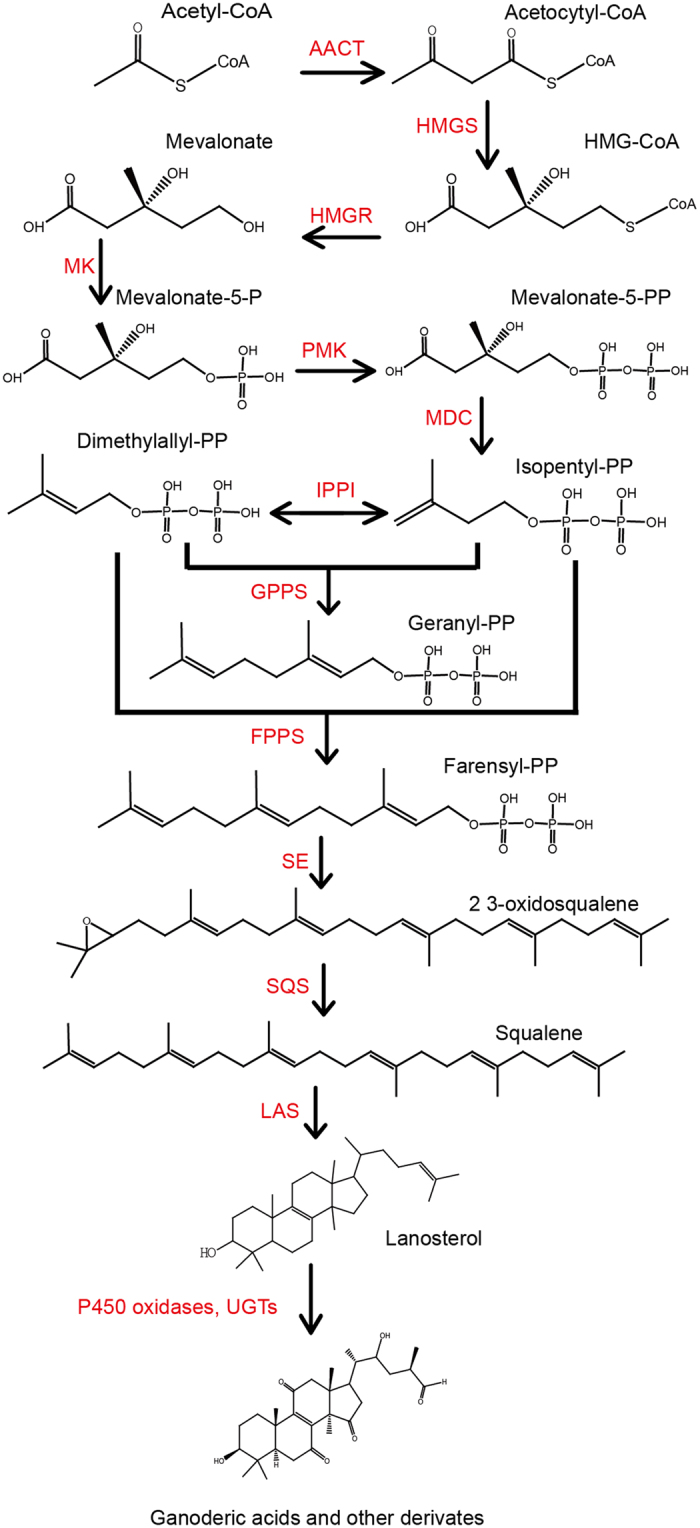
The biosynthetic pathway of ganoderic acids in *Ganoderma lucidum*. The enzyme’s acronyms used in the figure are: AACT: acetyl CoA: acetyl CoA C-acetyltransferase; HMGS: 3-hydroxy-3-methylglutaryl CoA synthase; HMG-CoA: 3-hydroxy-3-methylglutaryl CoA; HMGR: 3-hydroxy-3-methylglutaryl CoA reductase; MVA: mevalonate; MK: mevalonate kinase; IPPI: isopentenyl diphosphate isomer; FPP: farnesyl pyrophosphate; SS: squalene synthase; SE: squalene epoxidase; LAS: lanosterol synthase.; IPP: isopentenyl pyrophosphate; GPPS: geranyl diphosphate; GPP: geranyl pyrophosphate; FPPS: farnesyl diphosphate synthase.

**Figure 2:**
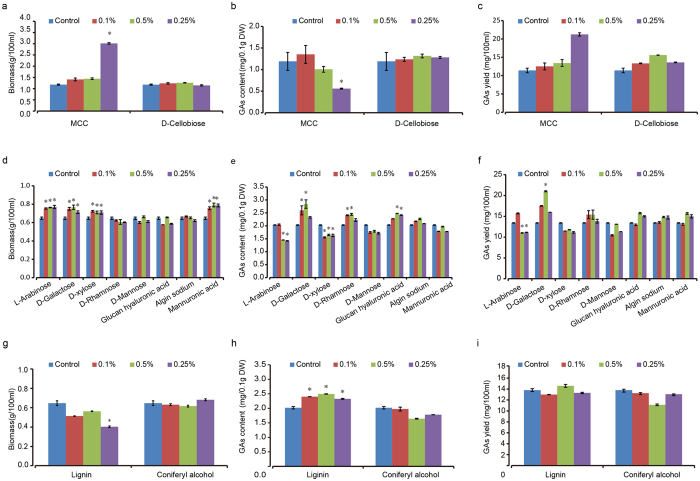
The effect of wood degradation compounds on the growth of *Ganoderma lucidum* and ganoderic acids biosynthesis in liquid fermentation. The effect of microcrystalline cellulose, hemicellulose, monosaccharides, lignin and conferral alcohol, each with different concentration, was studied on the biomass accumulation (**a,d,g**), GAs content in dry weight (**b,e,h**) and GAs total yield (**c,f,i**) in *G. lucidum* fermentation. Data were reported as the mean and standard error of the mean of at least three independent repetitions of each assay (Student’s test *p < 0.05). Error bars represent SD.

**Figure 3:**
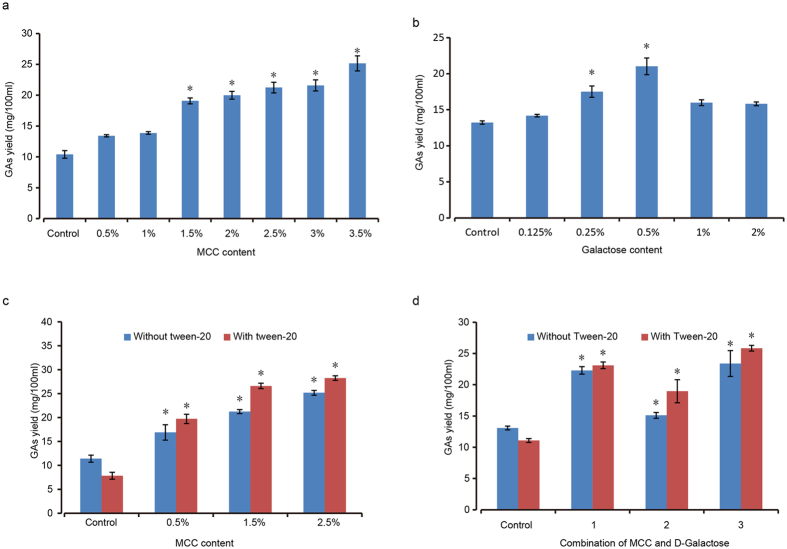
The effect of different concentrations of microcrystalline cellulose and D-Galactose on the ganoderic acids biosynthesis. The effect of different concentrations of MCC and D-Galactose on the GAs biosynthesis was tested separately (**a,b**). For comparison, the effect of Tween-20 on the GAs biosynthesis was also tested along with MCC and D-galactose (**c,d**). The effect of combining different proportion of MCC and D-Galactose on the GAs biosynthesis was evaluated as 1: MCC 1.5% + D-Galactose 0.25%; 2: MCC 0.75% + D-Galactose 0.5%; 3: MCC 1.5% + D-Galactose 0.5% (**d**). Data were reported as the mean and standard error of the mean of at least three independent repetitions of each assay (Student’s test *p < 0.05). Error bars represent SD.

**Figure 4:**
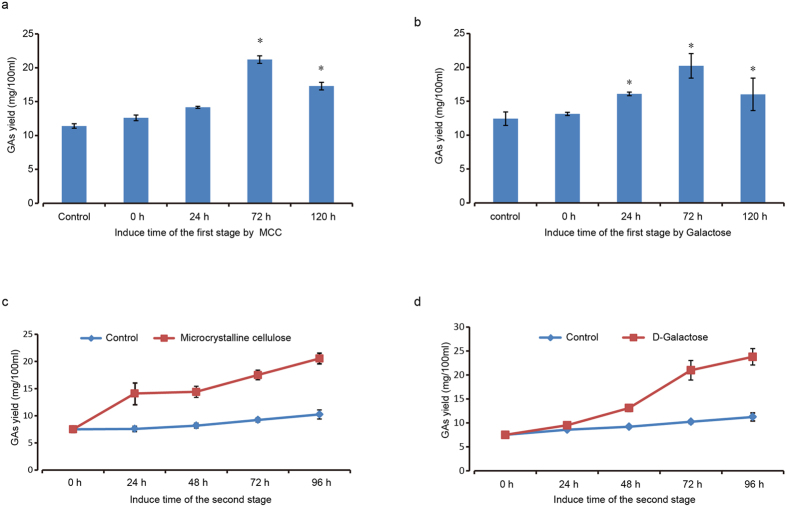
The time course of GAs yield induced by 1.5% microcrystalline cellulose and 0.5% D-Galactose. In the first stage, MCC (**a**) or galactose (**b**) were added at 0, 24, 72 and 120 h and all samples were collected for GAs quantification. For the yield optimization, *G. lucidum* culture with MCC (**c**) or galactose (**d**) adding at day 3 were further cultured as a second stage and the GAs yield were examined along the time course. Data were reported as the mean and standard error of the mean of at least three independent repetitions of each assay (Student’s test *p < 0.05). Error bars represent SD.

**Figure 5:**
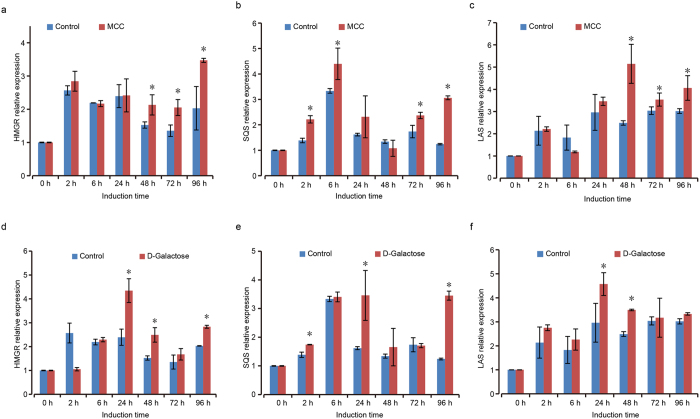
Transcription analysis of genes encoding enzymes for ganoderic acids biosynthesis in the presence of 1.5% microcrystalline cellulose or 0.5% D-Galactose. *G. lucidum* culture treated by MCC (**a**–**c**) or galactose (**d**–**f**) and the cells were retrieved at different time point for mRNA extraction. Quantitative RT-PCR was carried out with cDNA reverse transcribed from mRNA samples. HMGR: 3-hydroxy-3-methylglutaryl CoA reductase. SQS: squalene synthase. LAS: lanosterol synthase. Data were reported as the mean and standard error of the mean of at least three independent repetitions of each assay (Student’s test *p < 0.05). Error bars represent SD.

Previous studies showed that the yield of GAs can be improved by regulating the expression level of specific genes^47^,^51^. It is also reported that it was possible to increase the production of GAs by enhancing the expression level of a few genes in *G. lucidum* culture^51^,^52^. The biosynthesis of most triterpenoid derivatives shares a basic metabolic pathway, of which the final products are produced by a series of enzymatic reactions (Fig. 1). Thus investigation of the activity of the genes which are responsible for the tritepenoids synthesis may provide information at the molecular level. The qRT-PCR data showed that the activity of most genes was enhanced during MCC and galactose induction (Fig. 5).

SQS is a critical branch-point enzyme and an important regulatory point monitoring the carbon change into the terpenoid synthesis^53^,^54^. Studies showed that SQS was more critical for the GA biosynthesis than LAS and HMGR in *G. lucidum*^51^,^52^,^55^,^56^. Our data indicated that both MCC and galactose enhanced HMGR and SQS’s expression, while with different patterns (Fig. 5b,e). LAS, an enzyme that is close to the GAs production in the MVA biosynthesis pathway, also showed a different way of over-expression (Fig. 5c,f). It exhibited a tendency that MCC and galactose induced the above three genes’ expression. However, it is unknown that why a chemical could activate different gene’s expression. We hypothesized that MCC and galactose were unlikely used as nutrients during the fermentation. These two chemicals may globally affect the status of fungi growth. It correlates with the over-expression of three key genes for GAs production. We deduced that since these two chemicals were not direct substrate for GAs, the higher GAs production might come from elevated enzymes’ activity for its biosynthesis. Further studies may explore the mechanism how MCC and galactose induce the GAs enhanced production.

In all, we studied the influence of wood degradation components on GAs production in liquid cultivation of *G. lucidum* because the mushroom is capable to utilize the wood as sole nutrients. Although the saprophytic fungus is originally dependent on the timber for its life cycle, we found out that not all kinds of wood degradation components contributed to the GAs accumulation in the liquid culture. Our result showed that two possible wood degradation intermediates, MCC and a monosaccharide, the D-galactose, played a significant role in the secondary metabolism, and both chemicals increased the expression level of key genes that are responsible for the GAs biosynthesis. Both MCC and D-galactose are part of native cellulose structural components^57^,^58^. Currently it is hard to clearly explain why only a few components are strong inducers of secondary metabolites but this work provides a convenient, economical and safe approach for the enhancement of GAs. Our strategy paves a way to further explore the wood degradation process for the optimal production of GAs in *G. lucidum* and active components from other medicinal mushrooms.

## Methods

### Fungal strain and culture conditions

The *G. lucidum* dikaryotic strain CGMCC5.0026 was obtained from China General Microbiological Culture Collection Center (Beijing, China). Microcrystalline cellulose (MCC), lignin, D-cellobiose, D-galactose, L-arabinose, D-xylose and D-rhamnose were purchased from Sangon Biotech (Shanghai, China). Vegetative mycelia were grown on PDA plate at 28 °C in the dark. Liquid cultures were shaken at 28 °C with 125 revolutions per minute (rpm). Seed cultures were grown in 250 ml flasks containing 100 ml preculture medium (g/L: glucose 35, peptone 5, yeast extract 2.5, KH_2_PO_4_ 0.883, Vitamin B1 0.05, MgSO_4_·7H_2_O 0.5, initial pH 5.5) at 125 rpm and 28 °C for 7 d. The fermentation experiments were performed in 250 ml flasks containing 100 ml fermentation medium (g/L: glucose 35, peptone 5, yeast extract 5, KH_2_PO_4_ 0.883, Vitamin B1 0.05, MgSO_4_·7H_2_O 0.5, pH5.5) at 125 rpm at 28 °C for 7 days. All chemicals were added to the fermentation media at 72 h post of inoculation at indicated concentration. MCC and lignin were sterilized at 115 °C for 30 min. Instead, the rest of saccharide and sugars were sterilized by filtration through a 0.2 μm membrane.

### Measurement of GAs

After fermentation, *G. lucidum* cells were collected by centrifuge. The mycelium was then placed in a heated chamber at 50 °C for 4 h to dry. GAs were extracted from 0.1 g dried fungal mycelium by 50% (v/v) ethanol and measured as previously described 22,45,46,59. Briefly, 0.1 g mycelium powder was added with 3 ml 50% (v/v) ethanol for extraction. After centrifugal, the supernatants were dried at 50 °C under rotary evaporation. The residues were then resuspended with 3 ml water and extracted again with chloroform. After mixing, the chloroform layer was taken out and extracted with 5% (w/v) NaHCO_3_. The pH of the NaHCO_3_ layer was adjusted to 3.0 with 2 M HCl. Then the NaHCO_3_ layer is extracted with chloroform and dried at 40 °C under rotary evaporation. Finally precipitations were dissolved in anhydrous ethanol. The absorbance measured under 245 nm represents the GA yield.

### Transcriptional analysis

The mycelium samples were taken from fermentation media at indicated time. The mycelia were harvested by centrifuge and stored immediately in − 80 °C until use. Total RNA was extracted using RNAiso Kit (TaKaRa, Shanghai). The cDNA was synthesized with a HiFi Script Quick gDNA Removal Kit (TIANGEN Biotech., China). The RNA samples were quantified by a Nanodrop 2000 spectrophotometer (Thermo Scientific, USA). The quality of RNA was verified by electrophoresis. Fluorescence quantitative reverse transcription PCR (qRT-PCR) solution was prepared with SYBR Green qPCR Mix according to manual operation (Aidlab Biotech., China). The expression levels of 18 s, HMGR, SQS and LAS were analyzed using qPCR method. Primers were quoted from Chen^60^ (18s-F: TATCGAGTTCTGACTGGGTTGT; 18s-R: ATCCGTTGCTGAAAGTTGTAT); HMGR (F: GCGTCGGTAACATGATCCTT; R: GACAAGACTCCGCGAATAG); SQS (F: AAAACGCGACATTACCCAA; R: CTTGATGACCCCAGAGAAA); LAS (F: GAACCCGAAGCATTACAGGA; R: GAACCCACCGTCTGTGTTCT). The qRT-PCR was performed on a qTOWER 2.0 machine (Analytik Jena AG, Germany). The PCR reactions were set up by the following procedures: After an initial denaturation step at 94 °C for 3 min, amplification was carried out in three steps: 20 s of denaturing at 94 °C, 20 s of annealing at 54 °C and 30 s of extension at 72 °C for a total of 40 cycles. Identical PCR conditions were used for all targets.

### Statistical analysis

SPASS (version 19.0) was used to analyze data. Analysis of variance (ANOVA) of data and graphics was also generated by the software. Data were presented as mean ± standard error, and significant difference was analyzed at the 0.05 level by one-way ANOVA, LSD multiple range test.

## Additional Information

**How to cite this article:** Hu, Y. *et al*. Improved ganoderic acids production in *Ganoderma lucidum* by wood decaying components. *Sci. Rep.* **7**, 46623; doi: 10.1038/srep46623 (2017).

**Publisher's note:** Springer Nature remains neutral with regard to jurisdictional claims in published maps and institutional affiliations.

## References

[b1] Cao Y., Wu S.-H. & Dai Y.-C. Species clarification of the prize medicinal Ganoderma mushroom “Lingzhi”. Fungal Diversity 56, 49–62 (2012).

[b2] Lindequist U., Niedermeyer T. H. & Jülich W.-D. The pharmacological potential of mushrooms. Evidence-Based Complementary and Alternative Medicine 2, 285–299 (2005). 16136207 10.1093/ecam/neh107 PMC1193547

[b3] Boh B., Berovic M., Zhang J. & Zhi-Bin L. Ganoderma lucidum and its pharmaceutically active compounds. Biotechnology annual review 13, 265–301 (2007). 10.1016/S1387-2656(07)13010-6 17875480

[b4] Chang C. J. . Ganoderma lucidum reduces obesity in mice by modulating the composition of the gut microbiota. Nat Commun 6, 7489 (2015). 26102296 10.1038/ncomms8489 PMC4557287

[b5] Klupp N. L., Kiat H., Bensoussan A., Steiner G. Z. & Chang D. H. A double-blind, randomised, placebo-controlled trial of Ganoderma lucidum for the treatment of cardiovascular risk factors of metabolic syndrome. Sci Rep 6, 29540 (2016). 27511742 10.1038/srep29540 PMC4980683

[b6] Cui X. Y. . Extract of Ganoderma lucidum prolongs sleep time in rats. J Ethnopharmacol 139, 796–800 (2012). 22207209 10.1016/j.jep.2011.12.020

[b7] Wang P. Y., Zhu X. L. & Lin Z. B. Antitumor and Immunomodulatory Effects of Polysaccharides from Broken-Spore of Ganoderma lucidum. Front Pharmacol 3, 135 (2012). 22811667 10.3389/fphar.2012.00135 PMC3395810

[b8] Zhan L., Tang J., Lin S., Xu Y. & Qin C. Prophylactic Use of Ganoderma lucidum Extract May Inhibit Mycobacterium tuberculosis Replication in a New Mouse Model of Spontaneous Latent Tuberculosis Infection. Front Microbiol 6, 1490 (2015). 26779146 10.3389/fmicb.2015.01490 PMC4705449

[b9] Berger A. . Cholesterol-lowering properties of Ganoderma lucidum *in vitro, ex vivo*, and in hamsters and minipigs. Lipids in health and disease 3, 1 (2004). 14969592 10.1186/1476-511X-3-2 PMC385249

[b10] Hajjaj H., Macé C., Roberts M., Niederberger P. & Fay L. B. Effect of 26-oxygenosterols from Ganoderma lucidum and their activity as cholesterol synthesis inhibitors. Applied and environmental microbiology 71, 3653–3658 (2005). 16000773 10.1128/AEM.71.7.3653-3658.2005 PMC1168986

[b11] Lin S.-B., Li C.-H., Lee S.-S. & Kan L.-S. Triterpene-enriched extracts from Ganoderma lucidum inhibit growth of hepatoma cells via suppressing protein kinase C, activating mitogen-activated protein kinases and G2-phase cell cycle arrest. Life sciences 72, 2381–2390 (2003). 12639703 10.1016/s0024-3205(03)00124-3

[b12] Liu J. . Anti-androgenic activities of the triterpenoids fraction of Ganoderma lucidum. Food Chemistry 100, 1691–1696 (2007).

[b13] Sliva D. . Mushroom Ganoderma lucidum prevents colitis-associated carcinogenesis in mice. PLoS One 7, e47873 (2012). 23118901 10.1371/journal.pone.0047873 PMC3484149

[b14] Smina T. P., Joseph J. & Janardhanan K. K. Ganoderma lucidum total triterpenes prevent gamma-radiation induced oxidative stress in Swiss albino mice *in vivo*. Redox Rep 21, 254–261 (2016). 26817677 10.1080/13510002.2015.1126098 PMC6837450

[b15] Tung N. T. . Inhibitory effect on NO production of triterpenes from the fruiting bodies of Ganoderma lucidum. Bioorg Med Chem Lett 23, 1428–1432 (2013). 23357630 10.1016/j.bmcl.2012.12.066

[b16] Xuan M. . Chronic Treatment with a Water-Soluble Extract from the Culture Medium of Ganoderma lucidum Mycelia Prevents Apoptosis and Necroptosis in Hypoxia/Ischemia-Induced Injury of Type 2 Diabetic Mouse Brain. Evidence-Based Complementary and Alternative Medicine 2015 (2015). 10.1155/2015/865986 PMC4402482 25945116

[b17] Wagner R., Mitchell D. A., Lanzi Sassaki G., Lopes de Almeida Amazonas M. A. & Berovič M. Current techniques for the cultivation of Ganoderma lucidum for the production of biomass, ganoderic acid and polysaccharides. Food technology and biotechnology 41, 371–382 (2003).

[b18] Chen S. . Genome sequence of the model medicinal mushroom Ganoderma lucidum. Nature communications 3, 913 (2012). 10.1038/ncomms1923 PMC3621433 22735441

[b19] Fang Q. H. & Zhong J. J. Two‐stage culture process for improved production of ganoderic acid by liquid fermentation of higher fungus Ganoderma lucidum. Biotechnology progress 18, 51–54 (2002). 11822899 10.1021/bp010136g

[b20] Feng J. . A New Temperature Control Shifting Strategy for Enhanced Triterpene Production by Ganoderma lucidum G0119 Based on Submerged Liquid Fermentation. Appl Biochem Biotechnol 180, 740–752 (2016). 27272496 10.1007/s12010-016-2129-1

[b21] Zhu L.-W., Zhong J.-J. & Tang Y.-J. Significance of fungal elicitors on the production of ganoderic acid and Ganoderma polysaccharides by the submerged culture of medicinal mushroom Ganoderma lucidum. Process Biochemistry 43, 1359–1370 (2008).

[b22] Ren A. . Methyl jasmonate induces ganoderic acid biosynthesis in the basidiomycetous fungus Ganoderma lucidum. Bioresource technology 101, 6785–6790 (2010). 20395130 10.1016/j.biortech.2010.03.118

[b23] Ren A. . Transcript and metabolite alterations increase ganoderic acid content in Ganoderma lucidum using acetic acid as an inducer. Biotechnology letters 36, 2529–2536 (2014). 25216642 10.1007/s10529-014-1636-9

[b24] You B.-J. . A novel approach to enhancing ganoderic acid production by Ganoderma lucidum using apoptosis induction. Plos One 8, e53616 (2013). 23326470 10.1371/journal.pone.0053616 PMC3542374

[b25] Zhang G. . Ethylene promotes mycelial growth and ganoderic acid biosynthesis in Ganoderma lucidum. Biotechnol Lett 39, 269–275 (2017). 27771819 10.1007/s10529-016-2238-5

[b26] Xu Y.-N. & Zhong J.-J. Impacts of calcium signal transduction on the fermentation production of antitumor ganoderic acids by medicinal mushroom Ganoderma lucidum. Biotechnology advances 30, 1301–1308 (2012). 22036615 10.1016/j.biotechadv.2011.10.001

[b27] Xu Y. N., Xia X. X. & Zhong J. J. Induction of ganoderic acid biosynthesis by Mn2+ in static liquid cultivation of Ganoderma lucidum. Biotechnol Bioeng 111, 2358–2365 (2014). 24870062 10.1002/bit.25288

[b28] Li H. J. . Enhancement of ganoderic acid production by constitutively expressing Vitreoscilla hemoglobin gene in Ganoderma lucidum. J Biotechnol 227, 35–40 (2016). 27080449 10.1016/j.jbiotec.2016.04.017

[b29] Hirotani M., Asaka I. & Furuya T. Investigation of the biosynthesis of 3α-hydroxy triterpenoids, ganoderic acids T and S, by application of a feeding experiment using [1, 2-13C2] acetate. J. Chem. Soc., Perkin Trans. 1, 2751–2754 (1990).

[b30] Shiao M. S. Triterpenoid natural products in the fungus Ganoderma lucidum. Journal of the Chinese chemical society 39, 669–674 (1992).

[b31] Shang C.-H., Shi L., Ren A., Qin L. & Zhao M.-W. Molecular cloning, characterization, and differential expression of a lanosterol synthase gene from Ganoderma lucidum. Bioscience, biotechnology, and biochemistry 74, 974–978 (2010). 10.1271/bbb.90833 20460708

[b32] Xu J.-W., Zhao W. & Zhong J.-J. Biotechnological production and application of ganoderic acids. Applied microbiology and biotechnology 87, 457–466 (2010). 20437236 10.1007/s00253-010-2576-5

[b33] Zhao C. L., Cui X. M., Chen Y. P. & Liang Q. A. Key Enzymes of Triterpenoid Saponin Biosynthesis and the Induction of Their Activities and Gene Expressions in Plants. Natural product communications 5, 1147–1158 (2010). 20734961

[b34] Adaskaveg J., Gilbertson R. & Blanchette R. Comparative studies of delignification caused by Ganoderma species. Applied and environmental microbiology 56, 1932–1943 (1990). 16348229 10.1128/aem.56.6.1932-1943.1990 PMC184533

[b35] Baldrian P. & Valášková V. Degradation of cellulose by basidiomycetous fungi. FEMS microbiology reviews 32, 501–521 (2008). 18371173 10.1111/j.1574-6976.2008.00106.x

[b36] Hon D. N.-S. Cellulose: a random walk along its historical path. Cellulose 1, 1–25 (1994).

[b37] Lynd L. R., Weimer P. J., Van Zyl W. H. & Pretorius I. S. Microbial cellulose utilization: fundamentals and biotechnology. Microbiology and molecular biology reviews 66, 506–577 (2002). 12209002 10.1128/MMBR.66.3.506-577.2002 PMC120791

[b38] Davin L. B. & Lewis N. G. Lignin primary structures and dirigent sites. Curr Opin Biotechnol 16, 407–415 (2005). 16023847 10.1016/j.copbio.2005.06.011

[b39] Geib S. M. . Lignin degradation in wood-feeding insects. Proc Natl Acad Sci USA 105, 12932–12937 (2008). 18725643 10.1073/pnas.0805257105 PMC2529026

[b40] Zhang P., Chen F., Lai T. Q., Jin L. Y. & Li Y. [Effects of Loquat-Branch Dust Substitution on Ganoderma lucidum Cultivation in Its Main Active Components]. Zhong Yao Cai 38, 2464–2467 (2015). 27352526

[b41] Luna M. L., Murace M. A., Keil G. D. & Otaño M. E. Patterns of decay caused by Pycnoporus sanguineus and Ganoderma lucidum (Aphyllophorales) in poplar wood. IAWA Journal 25, 425–433 (2004).

[b42] Peláez F., Martínez M. J. & Martinez A. Screening of 68 species of basidiomycetes for enzymes involved in lignin degradation. Mycological research 99, 37–42 (1995).

[b43] Perilä O. The chemical composition of carbohydrates of wood cells. Journal of polymer science 51, 19–26 (1961).

[b44] Escalante-Chong R. . Galactose metabolic genes in yeast respond to a ratio of galactose and glucose. Proc Natl Acad Sci USA 112, 1636–1641 (2015). 25605920 10.1073/pnas.1418058112 PMC4321281

[b45] Wang W., Shang D. & Wen L. Quantitative analysis of triterpenoid in the mycelia of Ganoderma Lucidum. Edible Fungi of China 25, 30 (2006).

[b46] Tang Y.-J., Zhang W. & Zhong J.-J. Performance analyses of a pH-shift and DOT-shift integrated fed-batch fermentation process for the production of ganoderic acid and Ganoderma polysaccharides by medicinal mushroom Ganoderma lucidum. Bioresource technology 100, 1852–1859 (2009). 19010665 10.1016/j.biortech.2008.10.005

[b47] Ding Y.-X. . Molecular cloning, characterization, and differential expression of a farnesyl-diphosphate synthase gene from the basidiomycetous fungus Ganoderma lucidum. Bioscience, biotechnology, and biochemistry 72, 1571–1579 (2008). 10.1271/bbb.80067 18540102

[b48] Yang F.-C., Yang M.-J. & Cheng S. H. A novel method to enhance the mycelia production of Ganoderma lucidum in submerged cultures by polymer additives and agitation strategies. Journal of the Taiwan Institute of Chemical Engineers 40, 148–154 (2009).

[b49] Cheynier V., Comte G., Davies K. M., Lattanzio V. & Martens S. Plant phenolics: recent advances on their biosynthesis, genetics, and ecophysiology. Plant Physiology and Biochemistry 72, 1–20 (2013). 23774057 10.1016/j.plaphy.2013.05.009

[b50] Pusztahelyi T., Holb I. J. & Pócsi I. Secondary metabolites in fungus-plant interactions. Frontiers in plant science 6 (2015). 10.3389/fpls.2015.00573 PMC4527079 26300892

[b51] Shang C. H. . Cloning and characterization of a gene encoding HMG-CoA reductase from Ganoderma lucidum and its functional identification in yeast. Biosci Biotechnol Biochem 72, 1333–1339 (2008). 18460810 10.1271/bbb.80011

[b52] Zhao M. . Cloning and characterization of squalene synthase (SQS) gene from Ganoderma lucidum. Journal of microbiology and biotechnology 17, 1106 (2007). 18051320

[b53] Robinson G. W., Tsay Y., Kienzle B. K., Smith-Monroy C. A. & Bishop R. W. Conservation between human and fungal squalene synthetases: similarities in structure, function, and regulation. Molecular and Cellular Biology 13, 2706–2717 (1993). 8474436 10.1128/mcb.13.5.2706-2717.1993 PMC359645

[b54] Lee J.-H. . Cloning and expression of squalene synthase cDNA from hot pepper (Capsicum annuum L.). Molecules and cells 13, 436–443 (2002). 12132584

[b55] Xu J.-W., Xu Y.-N. & Zhong J.-J. Production of individual ganoderic acids and expression of biosynthetic genes in liquid static and shaking cultures of Ganoderma lucidum. Applied microbiology and biotechnology 85, 941–948 (2010). 19578843 10.1007/s00253-009-2106-5

[b56] Liang C.-X. . Enhanced biosynthetic gene expressions and production of ganoderic acids in static liquid culture of Ganoderma lucidum under phenobarbital induction. Applied microbiology and biotechnology 86, 1367–1374 (2010). 20077112 10.1007/s00253-009-2415-8

[b57] Klemm D., Philip B., Heinze T., Heinze U & Wagenknecht W. Text Book of Comprehensive Cellulose Chemistry. Volume 1, Fundamentals and Analytical Methods. (Wiley-VCH, Weinheim, 1998).

[b58] Atalla R. H. & Vanderhart D. L. Native cellulose: a composite of two distinct crystalline forms. Science 223, 283–285 (1984). 17801599 10.1126/science.223.4633.283

[b59] NA L., Liu X. H., Zhou J., Li Y. X. & Zhao M. W. Analysis of influence of environmental conditions on ganoderic acid content in Ganoderma lucidum using orthogonal design. Journal of microbiology and biotechnology 16, 1940–1946 (2006).

[b60] Chen J. . A real-time PCR method for the detection of Salmonella enterica from food using a target sequence identified by comparative genomic analysis. International journal of food microbiology 137, 168–174 (2010). 20060189 10.1016/j.ijfoodmicro.2009.12.004

